# Early spinal muscular atrophy treatment following newborn screening: A 20‐month review of the first Italian regional experience

**DOI:** 10.1002/acn3.52018

**Published:** 2024-04-10

**Authors:** Delia Gagliardi, Eleonora Canzio, Paola Orsini, Pasquale Conti, Vita Sinisi, Cosimo Maggiore, Maria Carla Santarsia, Giuseppina Lagioia, Giovanna Lupis, Isabella Roppa, Gaetano Scianatico, Daniela Mancini, Stefania Corti, Giacomo Pietro Comi, Mattia Gentile, Delio Gagliardi

**Affiliations:** ^1^ Dino Ferrari Center, Department of Pathophysiology and Transplantation University of Milan Milan Italy; ^2^ Pediatric Neurology Unit Pediatric Hospital “Giovanni XXIII” Bari Italy; ^3^ Medical Genetic Unit, Department of Reproductive Pregnancy Risk ASL BARI Bari Italy; ^4^ U.O.C. Medicina Fisica e Riabilitazione, A.O.U. Consorziale Policlinico di Bari Bari Italy; ^5^ Neuromuscular and Rare Disease Unit Fondazione IRCCS Ca' Granda Ospedale Maggiore Policlinico Milan Italy; ^6^ Neurology Unit Fondazione IRCCS Ca' Granda Ospedale Maggiore Policlinico Milan Italy

## Abstract

**Objectives:**

Mandatory newborn screening (NBS) for spinal muscular atrophy (SMA) was implemented for the first time in Italy at the end of 2021, allowing the identification and treatment of patients at an asymptomatic stage.

**Methods:**

DNA samples extracted from dried blood spot (DBS) from newborns in Apulia region were analysed for SMA screening by using a real‐time PCR‐based assay. Infants harbouring homozygous deletion of *SMN1* exon 7 confirmed by diagnostic molecular tests underwent clinical and neurophysiological assessment and received a timely treatment.

**Results:**

Over the first 20 months since regional NBS introduction, four out of 42,492 (0.009%) screened children were found to carry a homozygous deletion in the exon 7 of *SMN1* gene, with an annual incidence of 1:10,623. No false negatives were present. Median age at diagnosis was 7 days and median age at treatment was 20.5 days. Three of them had two copies of *SMN2* and received gene therapy, while the one with three *SMN2* copies was treated with nusinersen. All but one were asymptomatic at birth, showed no clinical signs of disease after a maximum follow‐up of 16 months and reached motor milestones appropriate with their age. The minimum interval between diagnosis and the treatment initiation was 9 days.

**Interpretation:**

The timely administration of disease‐modifying therapies prevented presymptomatic subjects to develop disease symptoms. Mandatory NBS for SMA should be implemented on a national scale.

## Introduction

Spinal muscular atrophy (SMA) is a relatively prevalent genetic disorder caused by biallelic mutations in *SMN1* gene and characterised by progressive degeneration of lower motor neurons. Primarily affecting infants and young children, its most severe and common form was once associated with early mortality prior to the advent of SMN augmenting therapy.^1^ Notably, nearly 50% of SMA cases are categorised as type 1, marked by a severe onset in early infancy and, in most cases, a presence of 2 *SMN2* copies. Without treatment, symptoms manifest within the first 6 months of life, accompanied by the inability to reach the milestones of sitting and walking. Most type 1 patients do not survive beyond the second year of life, often dying of respiratory failure unless mechanical ventilation is provided.^1^

A recent surge of innovative SMN‐augmenting therapies has prompted a major transformation in the landscape of SMA treatment, dramatically changing the natural history of the disease.^2^, ^3^, ^4^ FDA/EMA approvals have been granted to nusinersen^5^, ^6^ (Spinraza, an intrathecally administered antisense oligonucleotide), onasemnogene abeparvovec^7^, ^8^ (Zolgensma, a gene replacement therapy with a single intravenous delivery), and risdiplam^9^, ^10^ (Evrysdi, a small molecule with oral administration).

During the period of the present study, two of these treatments have secured approval in Italy for the presymptomatic treatment of genetically confirmed SMA patients. Onasemnogene abeparvovec targets all presymptomatic SMA patients carrying a genetic SMA diagnosis with two and three *SMN2* copies, with ongoing global deliberations concerning cases with four *SMN2* copies. Conversely, nusinersen can be administered to presymptomatic SMA patients of all ages. In the period of incidence, risdiplam was not yet approved for presymptomatic patients below 2 months of age (it was eventually approved in August 2023 for subjects with one to four SMN2 copies, regardless of the age).

The remarkable success of these interventions in clinical trials,^11^, ^12^, ^13^ with significantly better outcomes than those observed in symptomatic patients,^14^ has underscored the urgency of newborn screening (NBS) for SMA, which has received strong support from experts and a widespread distribution worldwide. Addressing patients during their presymptomatic phase holds immense potential to reshape the disease's trajectory, offering improved prognoses and enhancing overall quality of life. Traditionally, SMA diagnosis leaned heavily on clinical symptoms, often resulting in delayed intervention until irreversible damage was evident. Yet, advances in genetic screening have paved a path to early detection, offering a window for intervention before symptoms surface. This shift in the natural course of SMA signifies a pivotal stride towards proactive and more effective healthcare.

Across various nations, population‐based SMA‐NBS initiatives have been established.^15^ Typically involving DNA extraction from dried blood spot (DBS) samples, followed by real‐time quantitative polymerase chain reaction (qPCR) to ascertain homozygous exon 7 deletion of *SMN1* gene (>95% of SMA cases), these programs represent a significant step forward.^16^

Pioneering pilot studies have promoted the implementation of NBS for SMA in several countries and, in combination with the opportunity to treat in presymptomatic stage, have provided the first real‐world evidence of the efficacy of early treatment in SMA, prompting these initiatives to spread worldwide.^17^, ^18^, ^19^, ^20^, ^21^, ^22^

Apulia emerged as the pioneer Italian region to introduce mandatory SMA screening at birth, starting on 6 December 2021. A recent Italian pilot study on a 2‐year experience has shown that NBS has led to the identification of 15 newborns with a molecular diagnosis of SMA, enlightening the importance of standardisation of current molecular diagnostic techniques.^23^ This study aims to assess the impact of the implementation of the NBS in Italy on accessibility to care and patient outcomes, using the example of Apulia.

## Methods

Since the 6th of December 2021, infants born in one of the 26 birth centres in Apulia were screened for SMA as a part of NBS regional initiative. The samples were sent to the referral Medical Genetic Unit (ASL Bari) and a real‐time PCR‐based assay specific for the qualitative detection of the exon 7 *SMN1* gene (Eonis SMA kit, CE‐IVD, Revvity) was performed. Briefly, the molecular test used for SMA screening is based on target sequence‐specific primers and Taqman probes to amplify and detect *SMN1* exon 7 and *RPP30* as internal amplification control in the DNA extracted from DBS with Eonis DNA Extraction kit (CE‐IVD, Revvity). The analysis and interpretation of results was performed using Eonis analysis software (CE‐IVD, Revvity).

For presumptive screening positives infants, two diagnostic confirmation molecular tests were carried out on fresh blood samples only after obtaining parental consent: a real‐time PCR quantitative assay and the commercial AmplideX^®^ SMA Plus Kit (Asuragen), both used to confirm the homozygous deletion of *SMN1* exon 7 and to determine the number of copies of *SMN2*.

**Figure 1:**
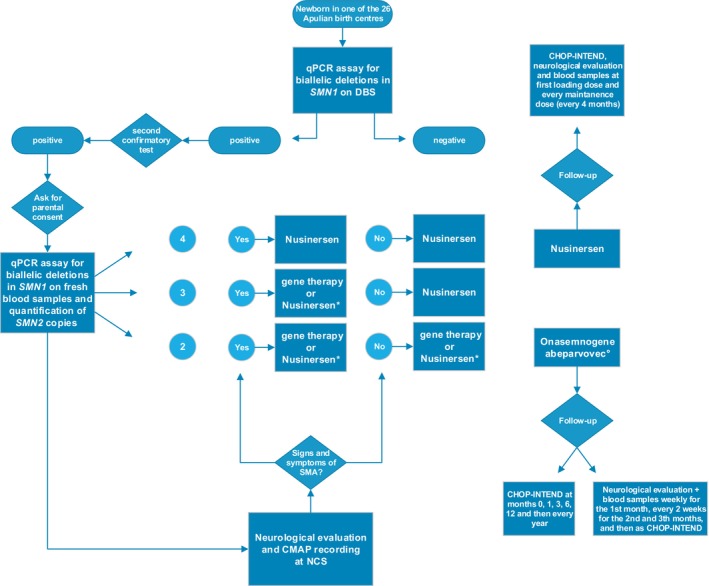
Workflow of diagnosis and treatment of SMA patients detected by newborn screening in Apulia region. Infants born in one of the 26 birth centres in Apulia are tested through a qPCR assay on dried blood spot (DBS) to look for biallelic deletions in the *SMN1* gene. If positive, a secondary confirmatory test is performed, and if again positive, a third test is performed after obtaining parental consent on fresh blood samples to look for biallelic deletions in *SMN1* gene and to quantify the number of *SMN2* copies. After diagnosis, a neurological evaluation and CMAP recording with stimulation at the patient's wrist are performed. Patients are eligible for different treatments depending on the presence of signs and symptoms of SMA and the number of *SMN2* copies. Patients with two copies are eligible for gene therapy or Nusinersen regardless of the presence of signs and symptoms. Patients with three copies are eligible for gene therapy or Nusinersen if symptomatic; if there are no signs or symptoms of SMA Nusinersen is the only treatment reimbursed by the Italian Medicines Agency (AIFA). Patients with four copies are only eligible for Nusinersen and are usually asymptomatic. For patients receiving Nusinersen, follow‐up includes CHOP‐INTEND administration, neurological evaluation and blood sampling at the first loading dose and at each maintenance dose (every 4 months). Patients treated with Onasemnogene abeparvovec will receive CHOP‐INTEND at months 0, 1, 3, 6, 12 and every year. In addition, neurological assessments and blood samples are taken weekly during the first month, every 2 weeks during the second and third months, and then at the same time points as CHOP‐INTEND. *Decision about treatment to be made with parents after explanations of risks and benefits. °After ruling out exclusion factors, including a positive anti‐AAV9 antibody titre.

All positive subjects received one of the two disease‐modifying therapies approved for presymptomatic SMA in Italy. Patients underwent motor nerve conduction study (NCS) before treatment to record baseline compound muscle action potential (CMAP) from stimulation at the wrist and were prospectively followed up with blood samples and neurological examination, including the CHOP‐INTEND scale. Figure 1 summarises the workflow for diagnosis and treatment of newborn with SMA (Fig. 1).

## Results

Over the initial 20‐month period following the introduction of regional NBS initiatives, the examination of 42,492 screened children revealed a positive result in four newborns (0.009% of the total), which was confirmed in all cases. This occurrence translated into an annual incidence rate of 1 in 10,623 births. Communication of the diagnosis and information about treatment options were led by a team consisting of a paediatric neurologist, a geneticist and a genetic counsellor. The parents of all four children granted their consent for confirmatory genetic testing and for participation in the study.

Demographic and clinical features of the four patients are shown in Table 1.

**Table 1:** Demographic and clinical features of children with homozygous deletion in *SMN1* identified by NBS.

	Patient 1	Patient 2	Patient 3	Patient 4
Gender	Female	Male	Male	Female
Current age	17 months	15 months	10 months	3 months
Family history	–	–	–	–
Number of *SMN2* copies	2	3	2	2
Age at diagnosis	7 days	7 days	9 days	7 days
CHOP‐INTEND at diagnosis/age	31/64 (15 days)	40/64 (17 days)	38/64 (13 days)	41/64 (8 days)
Ulnar CMAP at diagnosis/age	5.7 mV/17 days	5.8 mV/17 days	3.6 mV/13 days	4 mV/8 days
Clinical symptoms at birth	None	None	None	Generalised hypotonia, absent DTRs
Type of medication	Onasemnogene abeparvovec	Nusinersen	Onasemnogene abeparvovec	Onasemnogene abeparvovec
Age at first treatment	23 days	21 days^a^	20 days	16 days
Time from diagnosis to treatment	16 days	14 days	11 days	9 days
Best motor milestone achieved/age	Walking with support/15 months	Walking without support/14 months	Sitting without support/6.5 months	Head control/2.5 months
CHOP‐INTEND after treatment	64/64/12 months	64/64/6 months	64/64/6 months	35/64/3 months

^a^ Delay in treatment administration due to SARS‐CoV2 infection at 10 days old and lack of literature on potential contraindications.

**Figure 2:**
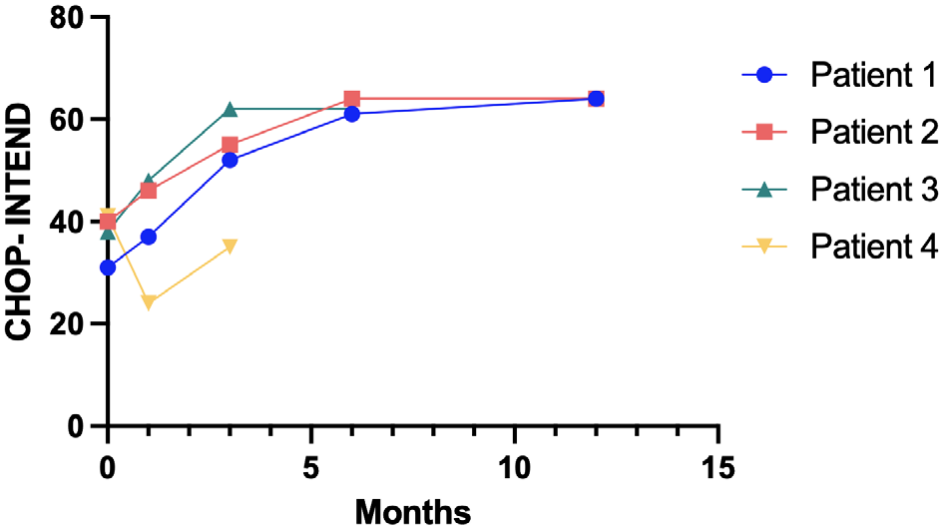
CHOP‐INTEND scores over time in newborn patients with SMA. CHOP‐INTEND scores at time of diagnosis and after 1, 3, 6 and 12 months.

The youngest patients were diagnosed at 7 days and the oldest one (patient 3) at 9 days, with a median age at diagnosis of 7 days. None of the patients, with the exception of one, had signs and symptoms of SMA at birth or during follow‐up and their neurological examination was unremarkable. All but one were found to carry two copies of *SMN2*, while one of them had three copies. Patient 4, carrier of 2 *SMN2* copies, displayed marked generalised hypotonia with a frog‐leg position and absent deep tendon reflexes (DTRs) at birth. All individuals underwent NCS and their ulnar CMAP was within the normal range (Fig. 2).Treatment decisions were made together with the parents, who were informed about the risks and benefits of each drug. After ruling out exclusion criteria (including a positive anti‐AAV9 antibody titre), patients 1, 2 and 4, who carried three copies of *SMN1* and were eligible for gene therapy, received a single injection of Onasemnogene abeparvovec. The earliest administration of gene replacement therapy occurred in patient 4 at 16 days of age, only 9 days after genetic diagnosis. Given the non‐refundability of Onasemnogene abeparvovec by the Italian Medicines Agency in asymptomatic carriers with three *SMN2* copies, patient 3 was treated with Nusinersen and received the first dose on the 20th day of life.

Overall, the median age at start of treatment was 20.5 days and the median time from diagnosis of 12.5 days, with the shortest interval between diagnosis and therapy being 9 days. This expedited treatment initiation occurred in the youngest patient in this cohort to undergo gene therapy (16 days).

All patients underwent a close follow‐up (Fig. 1), spanning up to 17 months. Patients 1, 2 and 3 remained asymptomatic throughout the follow‐up, achieved motor milestones appropriate for their age and reached the maximum score at CHOP‐INTEND scale at 12, 6 and 6 months, respectively. Patients with the longest follow‐up (patient 1 and 2) were able to walk within 15 months. Patient 3, who is currently 10 months old, could sit without support just after the 6th month of life. Neurological evaluation and CHOP‐INTEND scale administration in patient 4 showed a score of 35 at 3 months of age. The patient reached head control at 2.5 months of age.

## Discussion

The ability to diagnose SMA in its presymptomatic stages is a game changer, as it enables healthcare providers to initiate treatment strategies even before the first signs of neuromuscular weakness appear. This proactive approach capitalises on the potential to halt or slow down the progression of the disease, thereby improving motor function, extending survival rates and enhancing the overall quality of life for affected individuals and their families.

In this study, we reported on the results of implementation of the mandatory SMA‐NBS in the first Italian region, Apulia. Overall, the first 20 months following implementation of the regional NBS initiative yielded a minimal yet significant prevalence of homozygous *SMN1* deletions, with a disease annual incidence (1:10,623 newborns) consistent with literature epidemiological data.^24^ The screening test on DBS showed very high sensitivity and specificity, as all patients who tested positive were confirmed as true positives by genetic testing on fresh blood. To date, and since NBS approval, no clinical diagnosis of SMA 1 has been made in Apulia region, confirming the decisive role of screening in capturing the disease.

In our cohort, early administration of disease‐modifying therapies has allowed all the asymptomatic subjects to not develop any signs or symptoms of SMA and to achieve motor milestones appropriate for their age. Moreover, it is worth noticing that time to diagnosis and treatment is surprisingly short considering that sample collection, genetic testing and therapy administration took place in different centres. However, a longer follow‐up is needed to assess whether the timely delivery of gene therapy in the youngest and symptomatic patient was efficacious in preventing the development of further symptoms and in slowing down or halting disease progression. This patient presented at birth with a marked hypotonia, absent DTRs and frog‐leg like posture and she experienced a decrease in CHOP‐INTEND score between her birth and the third month of age. It can be speculated that this patient could have developed a severe form of SMA1 (e.g. SMA subtype 1A, according to the old clinical classification^25^) and early treatment likely slowed down progression of clinical symptoms.

While clinical classification based on symptom onset is not applicable to presymptomatic patients identified by NBS, the *SMN2* copy number plays a pivotal role, not only in guiding the access to therapy but also in stratifying the risk and timing of symptom onset and potentially predicting disease progression.

The vast majority of carriers of at least three copies of *SMN2* showed normal development when treated in the presymptomatic stages,^26^, ^27^ and less than 10% displayed a mild delay in motor development. Conversely, individuals with two *SMN2* copies have a more heterogeneous course. According to real‐word data, approximately half of them presented with symptoms at birth^27^ and, despite treatment, only a small proportion met age‐appropriate milestones. However, two‐thirds of individuals with two *SMN2* copies treated presymptomatically were able to walk, with a delay in reaching this milestone (after 18 months) for 29% of them.

In our cohort, the only patient with three *SMN2* copies had normal motor development and was able to walk independently by 14 months of age. Two of the three patients with two *SMN2* copies were asymptomatic and remained as such during follow‐up, without any motor delay. The one presenting with clinical symptoms had reached autonomous head control before 3 months of age, but follow‐up is still too short to predict her clinical course. Despite the small sample size, the current findings appear to be consistent with those of previous studies.^17^, ^18^, ^28^, ^29^

When comparing these data with those related to patients identified on the basis of clinical presentation and prior to the advent of NBS, it is clear that a striking difference exists in terms of outcomes. According to the natural history of SMA, only 34% of patients with three or more *SMN2* copies gain ambulation, and also when treated, they rarely walk independently if this milestone was not reached before the onset of symptoms.^30^

These findings highlight, once again, the relevance of a therapeutic window in neurodegenerative disorders,^31^ especially in those where a diagnosis can be made before symptom onset and that dispose of disease‐modifying therapies. Precocious treatment is of paramount importance to reduce severe disability, lowering costs on the health care system and to provide both better motor performances and improved life expectancy in these patients.

In recent years, genetic screening advancements have opened the possibility of early detection, creating an opportunity for intervention before symptoms appear. This represents a significant shift in SMA's natural progression, marking a crucial step toward more proactive and effective healthcare. Previously, SMA diagnosis relied on clinical presentation, often resulting in ineffective interventions to halt the neurodegenerative process. The immense potential of addressing patients during their presymptomatic phase holds tremendous promise for altering the disease's course, ultimately resulting in better outcomes and an overall improvement in the quality of life.

In this study, an impressive consent rate of 100% among parents demonstrated their keen interest in participation and this was attributed to the information received. Key contributors to the success of SMA‐NBS in Apulia were the existence of a mandatory NBS program, the presence of a neuro paediatric department able to accommodate the needs of both children and parents and the availability of a multidisciplinary team, including geneticists, paediatric neurologists, physiotherapists, anaesthesiologists, genetic counsellors, specialised nurses. This unique setup facilitated collaboration among experts, streamlined processes and accelerated the establishment of NBS programs.

Furthermore, treatment options for SMA patients diagnosed by NBS have been tailored based on a personalised approach. For patients eligible to both nusinersen and onasemnogene abeparvovec, the decision was made according to the severity of the disease, as determined by *SMN2* gene copy number, and the presence of potential contraindications, and in any case was taken after discussion with the patients' families.

Recognising the urgency of early treatment for severe SMA cases, efforts were directed towards refining the SMA‐NBS system. Although challenges exist, such as the timing of treatment initiation within the limited NBS framework, steps were taken to optimise the workflow. Educational initiatives aimed at parents and obstetricians were deemed crucial to enhancing awareness and expediting timely actions. This study revealed potential areas for improvement, such as shortening the timeline from birth to treatment decision.

We acknowledge the limitations of the small‐scale study with a relatively short follow‐up. However, the authors believe that also evidence from a small cohort of patients may add an important piece of knowledge about a rare disease like SMA.

In addition, the authors highlight the prospective design of this study and the availability of several clinical parameters, including motor scales and neurophysiological exams. All these measures are extremely helpful to gain a deeper understanding of the new shape of this disease in the current era characterised by early access to disease‐modifying therapies and they can potentially represent predictive tools in treated presymptomatic patients.^32^, ^33^

In conclusion, the successful start of SMA‐NBS study in Apulia illuminated the pathway towards early diagnosis and treatment over the country. While acknowledging its benefits, the study underscores the need for fairness in NBS programs, aiming to make SMA‐NBS mandatory across Italy. The journey ahead encompasses refining workflows, enhancing medical education and promoting wider awareness to ensure the swift and equitable implementation of SMA‐NBS throughout the nation. Moreover, the study recommended both immediate treatment and a monitored approach, aligning with evolving medical insights.

The advent of *SMN1‐*enhancing approaches has brought unprecedented advances to the world of neuromuscular diseases and the combination of disease‐modifying therapies with presymptomatic diagnosis has completely revolutionised the face of SMA.

## Author Contributions

DG: conceptualization; formal analysis; visualisation; writing—original draft preparation. EC: data curation; formal analysis; writing—original draft preparation. PO: investigation; formal analysis. PC: writing—review & editing. VS: data curation; writing—review & editing. CM: data curation; writing—review & editing. MCS: data curation; writing—review & editing. GL: investigation; formal analysis. GL: investigation; formal analysis. IR: investigation; formal analysis. GS: investigation; formal analysis. DM: investigation; formal analysis. SC: conceptualization; writing—review & editing. GPC: conceptualization; writing—review & editing. MG: investigation; formal analysis; writing—review & editing. DG: conceptualization; data curation; formal analysis; investigation; writing—review & editing.

## Conflict of Interest

The authors declare no existing conflict of interests.

## Ehics Approval

The local ethics committee Area 2 of the Paediatric Neurology Unit of the A.U.O. Consorziale Policlinico di Bari—Ospedale Pediatrico Giovanni XXIII approved the study. Written informed consent was obtained from all participants. The study was performed in accordance with the principles of the Declaration of Helsinki.

## Data Availability

The data that support the findings of this study are available from the corresponding author upon reasonable request.
